# Functional identification of microRNA-centered complexes in *C. elegans*

**DOI:** 10.1038/s41598-022-10771-2

**Published:** 2022-05-03

**Authors:** Shilpa Hebbar, Ganesh Panzade, Ajay A. Vashisht, James A. Wohlschlegel, Isana Veksler-Lublinsky, Anna Y. Zinovyeva

**Affiliations:** 1 Division of Biology, Kansas State University, Manhattan, 66506 USA; 2 Department of Biological Chemistry, David Geffen School of Medicine, University of California, Los Angeles, 90095 USA; 3 Department of Software and Information Systems Engineering, Ben-Gurion University of the Negev, 8410501 Beer-Sheva, Israel; 4 Present Address: Genomics Institute of the Novartis Research Foundation, San Diego, 92121 USA

**Keywords:** Genetics, Gene regulation

## Abstract

microRNAs (miRNAs) are crucial for normal development and physiology. To identify factors that might coordinate with miRNAs to regulate gene expression, we used 2′O-methylated oligonucleotides to precipitate *Caenorhabditis elegans* let-7, miR-58, and miR-2 miRNAs and the associated proteins. A total of 211 proteins were identified through mass-spectrometry analysis of miRNA co-precipitates, which included previously identified interactors of key miRNA pathway components. Gene ontology analysis of the identified interactors revealed an enrichment for RNA binding proteins, suggesting that we captured proteins that may be involved in mRNA lifecycle. To determine which miRNA interactors are important for miRNA activity, we used RNAi to deplete putative miRNA co-factors in animals with compromised miRNA activity and looked for alterations of the miRNA mutant phenotypes. Depletion of 25 of 39 tested genes modified the miRNA mutant phenotypes in three sensitized backgrounds. Modulators of miRNA phenotypes ranged from RNA binding proteins RBD-1 and CEY-1 to metabolic factors such as DLST-1 and ECH-5, among others. The observed functional interactions suggest widespread coordination of these proteins with miRNAs to ultimately regulate gene expression. This study provides a foundation for future investigations aimed at deciphering the molecular mechanisms of miRNA-mediated gene regulation.

## Introduction

Developmental and physiological processes require precise spatio-temporal regulation of gene expression. One post-transcriptional gene regulatory mechanism is directed by a class of small non-coding RNAs called microRNAs (miRNAs). miRNAs regulate a wide range of developmental and cellular processes, with dysregulated miRNA activity prevalent in diseases^1,2^. To exert their regulatory roles, miRNAs are loaded into Argonaute (AGO) proteins to form a miRNA Induced Silencing Complex (miRISC), which ultimately associates with an effector protein GW182. miRISC binding to the target mRNA via partial sequence complementarity between a miRNA and 3′ UTR of mRNA triggers a series of gene silencing mechanisms including translation inhibition, decapping, and mRNA decay^3,4^.

miRNAs are produced by a complex biogenesis process which involves enzymatic processing of miRNA intermediates in the nucleus and cytoplasm. Primary miRNAs are first cleaved by the Microprocessor complex (Drosha and DGCR8) to form pre-miRNAs^5,6^. After export from nucleus into cytoplasm, pre-miRNAs are further processed by Dicer to generate a miRNA duplex^7,8^. The miRNA duplex bound by Argonaute is then unwound, with the guide miRNA strand retained to form the mature miRISC, and the passenger strand released and degraded^9,10^. Each of the steps in miRNA biogenesis process can be regulated by RNA binding and other auxiliary factors thereby modulating the final gene-regulatory impact of miRNAs. These factors could bind miRNA intermediates or miRISC protein components to affect miRNA activity. For example, several RNA binding proteins including RBFOX3 and HnRNP A1 have been identified to bind to the hairpin structures of primary miRNAs and modulate their processing^11,12^. Other proteins, such as NHL-2 and CGH-1, associate with ALG-1 and AIN-1 to promote mRNA targeting^13^. RNA binding proteins Staufen^14^ and HuR^15,16^ indirectly affect miRNA-mediated gene silencing by competing for binding of the 3′UTRs of target mRNAs. Characterizing miRISC-associated protein complexes followed by functional analyses and mechanistic studies has high potential to identify additional mechanisms by which miRNA activity may be regulated.

Identifying proteins that associate with Argonaute proteins or miRNAs has been a productive approach to begin to unravel the mechanisms by which aspects of miRNA biogenesis and activity are regulated^17–20^. In human cells, investigation of proteomic profiles of AGO complexes led to identification of common protein interactors of all four AGO proteins, which included heat shock proteins, helicases, and components of translational machinery^17^. Some proteins identified in this study, including Hsc70/Hsp90 chaperone machinery, were later characterized for their roles in RISC loading of small RNA duplexes^21^. In mice, exploration of Dicer dependent and independent interactions of Ago2^18^ identified proteins that participate in miRISC-mediated decapping^22^, among other mechanisms. However, while proteomic approaches have characterized miRNA-associated complexes, it has been challenging to identify which of these co-factors are functionally important for miRNA activity, especially in tissue culture. In contrast, functional assays that quantitatively assess miRNA activity are available in model organisms such as *C. elegans*.

To identify miRNA/miRISC auxiliary cofactors important for miRNA gene regulatory activity, we took a functional proteomics approach. Specifically, we used 2′O-methylated biotinylated oligonucleotides to pull down three miRNAs of interest (let-7, miR-58, and miR-2) and subjected the associated protein complexes to proteomic analysis. Comparative analysis of miRNA pulldown and ALG-1 immunoprecipitation precipitates^19^ identified high confidence interactors common to all four datasets. In addition, we identified a unique set of interactors in each miRNA pulldown dataset. To assess whether the co-precipitated proteins are functionally important for miRNA activity, we performed RNAi knockdown of genes encoding for the putative physical interactors in multiple miRNA sensitized genetic backgrounds. Of the 39 interactors tested, depletion of 25 factors modified miRNA reduction of function phenotypes in one or more assays. Overall, we demonstrate that capturing physical interactors of miRNA machinery followed by in vivo functional assays is an efficient approach to identify novel players in miRNA-mediated gene regulation. While further mechanistic characterizations are necessary to determine the extent of the physical and functional interactions, this study identifies a functional requirement for a subset of potential ALG-1 and miRNA co-factors.

## Methods

### *C. elegans* maintenance, strains, and RNAi

All *C. elegans* strains were maintained on NGM and fed with *E. coli* OP50. Strains were maintained at 20 °C unless otherwise noted. RNAi knockdown was performed by feeding as previously described^23^.

The following strains were used in this study: N2 (wild type), MT7626 *(let-7(n2853)),* HW1113 *[Pdpy-30::GFP(PEST)-H2B::lin-41 3’ UTR (xeSi78); Pdpy-30::mCherry::H2B::artificial 3' UTR (xeSi36)]*, HW1114 *[Pdpy-30::GFP (PEST)-H2B::lin-41 3’ UTR (xeSi78); Pdpy-30::mCherry::H2B::artificial 3’ UTR (xeSi36), let-7(n2853)]*, VT1367 *(col-19::gfp (maIs105)),* VT1296 (*mir-48 mir-241(nDf51) col-19::gfp (maIs105)),* BW1932 *[hbl-1p::gfp::NLS::hbl-1 3’ UTR (ctIS39)]* and UY458 (*mir-48 mir-241(nDf51); hbl-1p::gfp::NLS::hbl-1 3*′* UTR (ctIS39)]),* OH812 (*otIs114 [Plim-6-gfp* + *rol-6(su1006)]),* OH3646 (*lsy-6(ot150); otIs114 [Plim-6-gfp* + *rol-6(su1006)])*, PS3662 *(syIs63[cog-1*::*gfp* + *unc-119(*+*)])*, OH7310 *(otIs193 [cog-1p*::*lsy-6* + *rol-6(su1006)] syIS63[cog-1*::*gfp* + *unc-119(*+*)]).*

### 2′O-Methyl oligo pulldowns and mass spec analysis

All experiments were performed on mixed-stage animals. Whole worm extracts^24^ and 2′O-methyl oligo pulldowns^25^ were performed as previously described. For mass spectrometry, each sample contained 20 mg of total protein. miRNA pulldowns were performed in two biological replicates using 2′O-methylated oligos with perfect complementation to miR-58, let-7, and miR-2, and scrambled oligo control (IDT). Sequences of the 2′O-methylated, biotinylated oligonucleotides are as follows: miR-58 oligo (5′-CAUCAUUGCCGUACUGAACGAUCUCAAGUC-3′), miR-2 oligo (5′-AUUCAGCACAUCAAAGCUGGCUGUGAUAUUCCA-3′), let-7 oligo (5′-UCUUCACUAUACAACCUACUACCUCAACCUU-3′), and scrambled oligo (5′-CAUCACGUACGCGGAAUACUUCGAAAUGUC-3′).

Mass spectrometric analysis of pulldown factors was performed as previously described^19^. Briefly, DTASelect^26^ was used to filter the proteins identified by applying a criterion that required proteins to have at least two unique peptides with total spectral intensities greater or equal to four in both replicates. To determine enrichment of protein association in a miRNA pulldown, the Normalized Spectral Abundance Factor (NSAF) values in miRNA pulldown were divided by that in control pulldown. NSAF value of zero in control was replaced by 1. Proteins with the pulldown/control ratio of ≥ 4 in all replicates were considered putative physical interactors.

### GO term and network analysis

Gene ontology analysis was performed using Database for Annotation, Visualization and Integrated Discovery (DAVID)^27^. Factors that had a fold change ≥ 4 in both replicates of the miRNA pulldowns were used for this analysis. For comparison, we included factors identified in atleast two replicates of ALG-1 IP for GO term analysis (as previously described^19^). Protein domain information, domain enrichment analysis, and the associated statistics were retrieved using STRING^28^. Enrichment for proteins harboring an RNA binding domain (RBD) among the proteins that passed our criteria was determined against a background set of *C. elegans* proteins that harbor the same RNA binding domain. Statistically significant enrichment was determined by applying Benjamini–Hochberg procedure on p-values to correct for multiple-testing. Network analysis was performed on the top 40 most enriched factors using STRING^28^ after excluding ribosomal proteins.

### Functional assays

#### let-7(n2853) vulval bursting assay

Vulval bursting assay was performed as previously described^29^. Briefly, *let-7(n2853)* and N2 worms were grown and maintained at 15℃. Embryos obtained through bleaching^30^ were plated on RNAi plates^23^ and grown until L4 larval stage. L4 animals were shifted to new RNAi plates and scored as day 1 adults for vulval bursting using a Leica dissecting microscope. Total number of worms (n) scored for this assay across two to four independent RNAi experiments ranged from 45 to 330.

#### col-19::gfp expression and seam cell number assay

*mir-48 mir-241(nDf51) col-19::gfp (maIs105)* animals were transferred to RNAi plates as L3 stage larvae and their progeny were scored for heterochronic phenotypes for hypodermal *col-19::gfp* expression. Worms with seam-only reporter expression were classified as having “delayed hypodermal *col-19::gfp* expression”. Seam cell numbers were scored by counting the number of seam cells expressing *col-19::gfp* between pharynx and anus. For most candidates, the total number of worms scored (n) across two to four replicates ranged from 22 to 80. For genes whose knockdowns resulted in severe developmental defects such as *snr-4, snr-6, let-363,* and *rnp-7,* the (n) was either 18 or 19.

#### lsy-6(ot150) ASEL cell fate assay

*lsy-6(ot150); plim-6::gfp* and *plim-6::gfp* worms were transferred onto RNAi plates as embryos. Their progeny were scored as L4s for specification of ASEL cell fate based on *plim-6::gfp* reporter expression. Worms lacking the reporter expression in ASEL neurons were scored as cell fate defective. Across two to four replicates, a total of 90 to 286 worms were scored.

#### pdpy-30::gfp::lin-41 reporter assay

*pdpy-30::GFP(PEST)-H2B::lin-41 3' UTR (xeSi78); pdpy-30::m Cherry::H2B::artificial 3' UTR (xeSi36)* and *pdpy-30::GFP (PEST)-H2B::lin-41 3' UTR (xeSi78); pdpy-30::mCherry::H2B::artificial 3' UTR (xeSi36), let-7(n2853)* embryos obtained by bleaching were plated on RNAi plates and grown at 15 °C. Reporter expression was measured in L4 stage animals by imaging the vulva at 63× magnification. To quantify expression levels in six vulval cells, ROIs were manually drawn and signal intensities within the ROI were measured using the Leica image analysis software. For each vulval cell, GFP signal intensity was divided by mCherry signal intensity and relative signal intensities were averaged across the six cells imaged in an individual animal. Representative images were equally adjusted after quantification to make the fluorescence more observable.

#### hbl-1p::gfp reporter assay

*hbl-1p::gfp::NLS::hbl-1 3′ UTR (ctIS39)* and *mir-48 mir-241(nDf51); hbl-1p::gfp::NLS::hbl-1 3′ UTR (ctIS39)* embryos were obtained by bleaching. Embryos were transferred to RNAi plates and animals were scored for *hbl-1p::gfp* expression in hypodermal cells at early to mid L3 stage. Animals were staged by time of development, and gonad size and shape.

#### Uterine cog-1 reporter expression

*cog-1::gfp* and *pcog-1::lsy-6; cog-1::gfp* animals were transferred onto RNAi plates as embryos and their F1 progeny were scored at L4 stage for *cog-1::gfp* expression in the uterine cells. Worms expressing *cog-1::gfp* in both uterine cells and vulval cells were scored as wild type. Worms that lacked *cog-1::gfp* reporter expression in either of the two uterine cells were scored as abnormal.

### Fluorescence microscopy, image capture and illustrations

**Figure 1:**
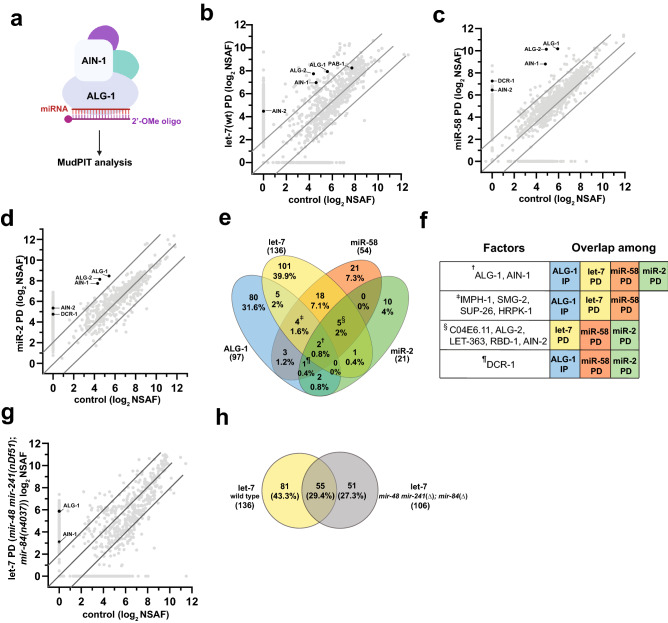
miRNA pulldowns (PDs) identify putative physical interactors of miRNA complexes. (**a**) Protein complexes associated with miRNAs were isolated using 2′-OMe modified oligonucleotides complementary to the miRNA of interest and subjected to MudPIT mass spectrometry analysis (drawn using BioRender (biorender.com)). (**b**–**d**) Average Normalized Spectral Abundance Factor (NSAF) values of factors identified in pulldown experiments (Y-axis) are plotted against that of corresponding scrambled oligo controls (X-axis) for (**b**) let-7 PD, (**c**) miR-58 PD and (**d**) miR-2 PDs. Highlighted in black are key miRISC components and components of miRNA biogenesis machinery. (**e**) Venn diagram showing the number of factors that passed a set of criteria to qualify as a putative interactor (for description of criteria, see “Methods”). Percentages shown here are percentage of total number of proteins (308) that passed the criteria in interaction datasets including ALG-1^19^. Proteins commonly co-precipitated with ^**†**^ALG-1, let-7, miR-58, and miR-2, ^**ǂ**^ALG-1 IP, let-7 PD, and miR-58 PD, ^**§**^let-7 PD, miR-58 PD, miR-2 PD, and ^**¶**^ALG-1 IP, miR-58 PD, and miR-2 PD. (**f**) Factors commonly identified in two or more interaction datasets include core miRNA machinery components. Symbols in the left column correspond to overlapping protein populations in (**e**). Colored boxes show which IP or PD precipitated the proteins listed in the left column. (**g**) NSAF values of proteins identified in pulldowns with let-7 complementary oligonucleotide from *mir-48 mir-241(nDf51); mir-84(n4037)* mutant animals, plotted against NSAF of proteins identified in scrambled oligo control. (**h**) let-7 complementary oligonucleotide precipitates a partially overlapping set of factors from *let-7*-family miRNA deletion backgrounds.

Fluorescence equipped Zeiss Axioplan 2 or Leica DM6 upright microscopes were used for scoring phenotypes. Images were captured using the Leica DM6B camera and processed using the Leica Application Suite X (3.4.1.17822) software (https://www.leica-microsystems.com/products/microscope-software/p/leica-las-x-ls/). Illustrations in Figs. 1a and 6e–g were drawn using BioRender (biorender.com).

### Statistical analysis

All statistics were done using GraphPad Prism (9.2.0 (332)) software. Statistical significance was determined using a one-way ANOVA test with predetermined comparisons. Bonferroni correction was applied as a post hoc analysis. T-test was used to determine statistical significance of *pdpy-30*::*gfp::lin-41, hbl-1p::gfp,* and *cog-1::gfp* reporter assays.

## Results

### miRNA pulldowns (PDs) identify overlapping sets of putative physical interactors of miRNA-centered complexes

To identify factors that may regulate miRNA activity, we sought to determine the molecular composition of protein complexes associated with let-7*,* miR-58, and miR-2 miRNAs. *let-7* is highly conserved across all bilateral animals^31^ and is required for the larval to adult transition in *C. elegans*^32^. miR-58 is a highly abundant miRNA that regulates lifespan and dauer formation^33^, primarily by coordinating with the TGF-β pathway^34,35^. miR-2, a neuronal miRNA conserved among invertebrates^36^, is necessary for proper neuromuscular junction function in *C. elegans*^37^. We used biotinylated, 2′O-methylated oligonucleotides with perfect sequence complementarity to mature miRNA sequences to pulldown miRNAs of interest and characterized the precipitates using a shotgun proteomics approach (Fig. 1a) to identify proteins associated with miRNAs of interest compared to scrambled control (Fig. 1b–d, Supplementary Table S1). To identify high confidence interactors, we retained only the proteins that were ≥ fourfold enriched in miRNA pulldowns over the scrambled control, had a minimum NSAF value of 4, and were identified in all replicates. Overall, a total of 211 proteins passed the criteria we set (Fig. 1e), with 136 factors co-precipitating with let-7, 54 factors co-precipitating with miR-58, and 25 factors co-precipitating with miR-2 (Fig. 1e, Supplementary Table S1). Among the proteins enriched in miRNA co-precipitates were known miRISC components ALG-1 and ALG-2, the two major miRNA-associated Argonautes in *C. elegans*^38^ and AIN-1 and AIN-2, GW182 homologs and miRISC effectors^39,40^ (Fig. 1b–f). In addition, DCR-1 nuclease, responsible for pre-miRNA processing, was detected in all pulldown experiments performed, but did not meet our stringent interaction criteria in the let-7 pulldown (Fig. 1e,f, Supplementary Table S1).

To determine the overlap between complexes precipitated by miRNA pulldowns and those previously found to associate with ALG-1^19^, we compared miRNA and ALG-1 co-precipitated factors (Fig. 1e,f, Supplementary Table S1). Eleven (8%) let-7 interactors, ten (18.5%) miR-58 interactors, and five (24%) miR-2 interactors were found to overlap with the ALG-1 co-immunoprecipitated dataset (Fig. 1e, Table 1). Overall, 41 proteins were present in at least two interaction datasets (Fig. 1e,f, Table 1), potentially representing general miRNA-associated co-factors. In addition, four proteins, HRPK-1, SMG-2, IMPH-1, and SUP-26, were present in 2 out of 3 pulldowns and the ALG-1 IP (Fig. 1e,f, Table 1). Their homologs were also found to co-immunoprecipitate with human and/or mouse Argonautes^17–19^, suggesting that they may have a conserved function in miRNA-mediated gene regulation. In fact, we previously confirmed a physical HRPK-1 interaction with ALG-1 and reported *hrpk-1* to genetically interact with multiple miRNAs^41^. Five proteins were commonly captured in all the miRNA pulldowns (Fig. 1e,f, Table 1). Interestingly, one such protein was LET-363, an mTOR homolog^42^ (Fig. 1e,f, Table 1). Similarly, we observed overlaps between our miRNA co-precipitates and previously reported miRNA physical interactors^43,44^ (Supplementary Table S2). Overlaps among our miRNA interaction datasets and AIN-1 and AIN-2 co-precipitates^40,44^ were also observed, further emphasizing that our approach captured potential miRISC interactors (Supplementary Table S2). Finally, candidates identified in genetic screens for miRNA and siRNA pathway genes also intersected with many of our miRNA co-precipitates^29,45,46^ (Supplementary Table S2). The observed overlaps among various groups of physical and genetic interactors support the idea that we are detecting real physical interactors of miRNA-centered complexes.

**Table 1:** Proteins that co-precipitated with two or more miRNAs or with ALG-1.

Sequence name	Protein name	Average of NSAF ratios_pulldown/control_ (spectral count)				Description
		let-7	miR-58	miR-2	ALG-1^19^	
F48F7.1	ALG-1	6 (88)	19 (328)	8 (73)	573 (229)	Argonaute, miRISC component
C06G1.4	AIN-1	5 (25)	16 (83)	11 (28)	125 (122)	miRISC component (GW182 homolog)
C04E6.11	C04E6.11	6 (86)	5 (35)	5 (7)	NA	Unknown
T07D3.7	ALG-2	115 (70)	293 (292)	105 (47)	NA	Argonaute
B0261.2	LET-363	5 (8)	9 (8)	47 (24)	NA	*C. elegans* Mtor
T23F6.4	RBD-1	20 (124)	5 (55)	27 (28)	NA	rRNA processing
M88.5	IMPH-1	9 (310)	10 (237)	NA	23 (39)	KH domain, RNA binding protein
F26B1.2	HRPK-1	146 (19)	44 (4)	NA	8 (14)	KH domain, RNA binding protein
R10E4.2	SUP-26	139 (20.5)	43 (5)	NA	113 (29)	Translational regulation
Y48G8AL.6	SMG-2	6 (43)	45 (17)	NA	9 (4)	NMD protein
B0041.2	AIN-2	22 (6)	87 (83)	NA	NA	miRISC component
Y49E10.15	SNR-6	7 (41)	5 (19)	NA	NA	Small nuclear ribonucleoprotein
Y71F9B.4	SNR-7	8 (42)	260 (14)	NA	NA	Small nuclear ribonucleoprotein
W08E3.1	SNR-2	9 (48)	5 (19)	NA	NA	Small nuclear ribonucleoprotein
Y116A8C.42	SNR-1	10 (55)	4 (10)	NA	NA	Small nuclear ribonucleoprotein
C52E4.3	SNR-4	381 (31)	4 (13)	NA	NA	Small nuclear ribonucleoprotein
F43H9.3	F43H9.3	4 (23)	52 (8)	NA	NA	Predicted to enable nucleotidyltransferase activity
ZC373.2	ZC373.2	56 (5)	43 (5)	NA	NA	Unknown
W07E6.1	NSUN-1	12 (5)	67 (12)	NA	NA	Nop2 (NOP2)/SUN domain family member
Y38C9A.2	CGP-1	61 (22)	52 (8)	NA	NA	Predicted to enable GTPase activity
K10D2.3	CID-1	16 (53)	14 (6)	NA	NA	RNA 3′ uridylation
W05F2.6	W05F2.6	139 (21)	114 (14)	NA	NA	Unknown
T01H10.8	LYST-1	41 (38)	15 (12)	NA	NA	Lysosomal trafficking regulator protein
K04G7.10	RNP-7	36 (14)	66 (5)	NA	NA	RNA binding protein
Y37H2A.1	Y37H2A.1	147 (25)	65 (8)	NA	NA	Predicted to enable hydrolase activity
F56B3.5	ECH-5	94 (20)	64 (12)	NA	NA	Enoyl-CoA hydratase
F57H12.6	F57H12.6	166 (12)	4 (9)	NA	NA	Unknown
F42A6.7	HRPA-1	30 (5)	14 (4)	NA	NA	RNA binding protein
F25B5.7	NONO-1	84 (14)	100 (13)	NA	NA	Conserved nuclear protein
H20J04.8	MOG-2	161 (34)	NA	114 (6)	NA	Enables U2 snRNA binding activity
W02F12.5	DLST-1	88 (75)	NA	NA	18 (12)	DihydroLipoamide *S*-SuccinylTransferase
F33D11.10	F33D11.10	16 (7)	NA	NA	29 (4)	RNA helicase activity
ZC434.5	EARS-1	14 (9)	NA	NA	7 (4)	Glutamate-tRNA ligase activity
H05C05.1	H05C05.1	32 (9)	NA	NA	12 (4)	Predicted to enable RNA strand annealing activity
Y47D3B.10	DPY-18	NA	15 (9)	NA	23 (5)	Procollagen-proline 4-dioxygenase activity
K02F2.1	DPF-3	NA	58 (14)	NA	42 (15)	Serine-type peptidase
F52B5.3	F52B5.3	6 (40)	NA	NA	6 (4)	Predicted to enable ATP binding activity
F18H3.3	PAB-2	NA	20 (9)	NA	5 (69)	Poly-A-binding protein
K12H4.8	DCR-1	NA	153 (84)	27 (10)	6 (5)	Small RNA processor
F37C12.11	RPS-21	NA	NA	89 (4)	39 (33)	Ribosomal protein
Y71F9AL.9	Y71F9AL.9	NA	NA	45 (6)	37 (7)	Unknown

The table shows the proteins that co-precipitated with either two or more miRNAs in our study and/or identified in previously reported ALG-1 IP^19^.

Due to a high level of sequence similarity amongst the *let-7* miRNA family members, the *let-7* complementary oligonucleotide precipitates other members of the miRNA family, albeit with reduced efficiency^47^. To determine whether distinct populations of proteins might associate with *let-7* miRNA family members, we performed additional *let-7* pulldown experiments in *mir-48 mir-241(nDf51); mir-84(n4037)* mutant animals (Fig. 1g, Supplementary Table S1). 55 (29.4%) proteins were in common among both *let-7* pulldowns, suggesting that these factors may interact with let-7 itself (Fig. 1h). The 51 (27.3%) proteins that precipitated with the let-7-complementary oligonucleotide from the *mir-48 mir-241(nDf51); mir-84 (n4037)* animals may similarly represent let-7-interacting factors, having been enriched in the let-7 pulldown in the absence of miR-48, miR-241, and miR-84 (Fig. 1h). In contrast, 81 (43.3%) proteins were present only in wildtype background pulldowns (Fig. 1h), suggesting that these factors may normally associate with miR-48, miR-241 and miR-84 (Fig. 1h). While we cannot rule out an association with the remaining let-7 family miRNAs, miR-793–795, the low relative abundance of these miRNAs^19^ suggests that miR-793–795 interactors are unlikely to represent significant fractions of the observed co-precipitates. Finally, some co-precipitates could represent non-specific interactions.

### Ribonucleoprotein complex components are enriched among miRNA interactors

**Figure 2:**
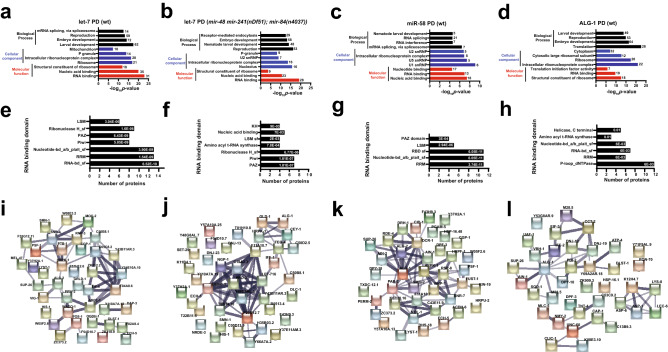
miRNA pulldowns identify components of translation machinery and mRNA processing factors, which may form a functional network. (**a**–**d**) Terms identified through GO analysis for biological processes (top four), cellular components (top four) and molecular function categories (top three) among let-7 PDs in (**a**) wild type, (**b**) *mir-48 mir-241(nDf51); mir-84(n4037)* backgrounds, (**c**) miR-58 PD and (**d**) ALG-1 IP^19^. Total number of proteins classified under each term shown adjacent to respective bars. (**e–h**) RNA binding domains identified in factors captured in let-7 PDs from (**e**) wild type, and (**f**) *mir-48 mir-241(nDf51); mir-84(n4037)*) backgrounds, (**g**) miR-58 PD and (**h**) ALG-1 IP. The FDR adjusted p-values for enrichment of proteins harboring individual domains are shown within the respective bars. (**i**–**l**) The reproducibly enriched proteins form functional network. Network analysis was performed using STRING^28^ on top 40 enriched interactors in (**i**) let-7 PD (wild type), (**j**) let-7 PD (*mir-48 mir-241(nDf51); mir-84(n4037))* (**k**) miR-58 PD and (**l**) ALG-1 IP. Ribosomal proteins were excluded from this analysis. Thickness of edges represents degree of confidence of functional linkages. The number of edges is significantly higher than expected, with a *p*-value < 1.0e−16.

To understand what biological processes and functions are represented in the miRNA-precipitated complexes, we performed Gene Ontology (GO) analysis on putative miRNA interactors (Fig. 2a–d, Supplementary Table S3). Factors implicated in embryonic and larval developmental processes were commonly enriched in all interaction datasets (Fig. 2a–c; ALG-1 interactome analysis is shown in Fig. 2d for comparison^19^, Supplementary Table S3). Selective enrichment for splicing associated factors was observed in let-7 and miR-58 PD datasets (Fig. 2a,c, Supplementary Table S3). Components of intracellular ribonucleoprotein complexes were consistently captured in all datasets (Fig. 2a–d and Ref.^19^). Enrichment for ribosomal components was observed only in let-7 and ALG-1 datasets (Fig. 2a,b,d and Ref.^19^). Unsurprisingly, the RNA/nucleic acid binding term was commonly overrepresented in all the datasets in the molecular function category (Fig. 2a–d and Ref.^19^). miR-2 interactors did not show an enrichment for any of the GO terms (Supplementary Table S3), potentially due to the low number of interactors captured in our pulldown and were therefore excluded from further analysis.

As RNA binding proteins (RBPs) were enriched in miRNA co-precipitates (Fig. 2a–d) and RBPs carry distinct domains critical for their RNA binding activity, we examined what RNA binding domains (RBDs) were present in proteins identified in our pulldowns and ALG-1 IP (Fig. 2e–h, Supplementary Table S3). At least 5 different RBDs were observed among RBPs in all the datasets. Notably, RBDs such as RNA Recognition Motif, KH, PAZ, and Nucleotide-binding alpha–beta plait domain superfamily were present in RBPs in one or more datasets (Fig. 2e–h, Supplementary Table S3). Overall, miRNA pulldowns captured factors that may play critical roles during the lifecycle of RNA.

To determine whether factors identified through our proteomics approach form a functional network, we performed network analysis using STRING^28^ (Fig. 2i–l). STRING predicts candidate protein interactions by utilizing both known and predicted protein–protein interactions sourced from databases, text mining, experimental, and co-expression data^28^. Top 40 enriched putative interactors in each dataset, minus the ribosomal proteins, were chosen for this analysis. Interestingly, we observed that in all the datasets proteins formed functional networks with a significant number of edges (*p*-value < 1.0e−16, as determined by STRING) (Fig. 2i–l), further supporting the idea that miRNA pulldown-captured proteins form functional complexes that may coordinate with miRNAs to regulate gene expression.

### Functional analysis of putative miRNA interactors

To identify which putative interactors might functionally coordinate with miRNAs to regulate gene expression, we took advantage of sensitized genetic backgrounds with reduced miRNA or miRNA family activity. These functional assays with quantifiable phenotypic outputs allow for assessment of a gene’s role in miRNA-mediated gene repression. Our pulldown experiments targeted miRNAs with varied functions and spatio-temporal expression patterns. Some of the identified interactors of miRNA- or ALG-1-centered complexes could have broad functional requirements, while others could be specific to a particular tissue or a developmental time. We hypothesized that knockdown of generally-required factors in multiple sensitized miRNA backgrounds would modulate phenotypes in multiple functional assays. In contrast, spatio-temporal specificity of the putative interactors may limit their functional relevance to specific miRNAs and may not result in a phenotype in some, or all, of our assays. In addition, the miRNA-centered protein complex analyses potentially identified interactors that may positively or negatively modulate microRNA activity. Knockdown of these factors in sensitized genetic backgrounds may therefore result in an enhancement or a suppression of the phenotype associated with reduction of miRNA function.

For our functional assessment, we prioritized factors that were highly enriched in our pulldown and/or ALG-1 IP experiments^19^ and were captured in multiple datasets. We excluded ribosomal proteins and factors lacking RNAi clones. The 39 candidates assayed ranged from common interactors of miRNA(s) and ALG-1 (6), common miRNA interactors (6), ALG-1 interactors (16), and specific miRNA interactors [let-7 (10), and miR-58 (1)] (Supplemental Table S4). Among the ALG-1 interactors, we assayed genes encoding for six proteins consistently identified in human and mouse AGO IP (referred to as conserved AGO interactors from hereon).

#### RNAi knockdown of genes of let-7 and ALG-1 interactors alters let-7(n2853) mutant phenotype

**Figure 3:**
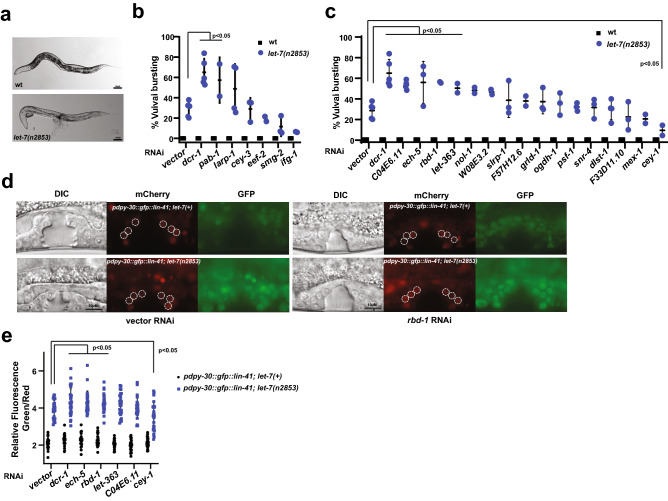
RNAi knockdown of genes encoding ALG-1 and let-7-associated factors genetically modifies *let-7(n2853)* vulval bursting phenotype. (**a**) *let-7(n2853)* mutants show partially penetrant vulval bursting phenotype at 15 °C. RNAi of (**b**) genes of previously reported conserved interactors of AGO^19^ and (**c**) let-7-precipitated factors in wild type and *let-7(n2853)* mutants. Each dot represents an independent RNAi experiment. Statistical significance was determined by one-way ANOVA with post hoc Bonferroni correction. (**d**) Effects of vector and *rbd-1* RNAi on *pdpy-30::gfp::lin-41* 3′UTR reporter expression in the vulval cells of wild type and *let-7(n2853)* L4 larvae. Compromised miRNA activity in *let-7(n2853)* background leads to de-repression of *pdpy-30::gfp::lin-41 3′ UTR* reporter expression levels (quantified in **e**). (**e**) RNAi of genes of modifiers of *let-7(n2853)* vulval bursting phenotype affects *pdpy-30::gfp::lin-41 3ʹUTR* reporter expression levels in *let-7(n2853)* background. Each dot represents the relative intensity in an individual worm. Vulval precursor cells used for fluorescence quantification are highlighted with dashed circles. T-test was used to determine statistical significance. Vector = empty vector RNAi control.

let-7 is essential for *C. elegans* development and promotes transition from the fourth larval stage (L4) to adulthood^32^. Loss of *let-7* function results in vulval bursting and failure of seam cells to differentiate during the L4 to adult transition^32^. *let-7(n2853)* is a temperature-sensitive reduction of function mutation that impairs regulation of let-7 targets, including *lin-41*^48^. *let-7(n2853)* mutants have a partially penetrant vulval bursting phenotype at permissive temperature^32^ (15 °C) (Fig. 3a). To determine whether the identified let-7 and ALG-1 interactors are functionally important for let-7 miRNA activity, we used this well-established genetic background to assay the effects of gene knockdown on *let-7(n2853)* bursting phenotype*.* RNAi of six genes enhanced vulval bursting of *let-7(n2853)* mutant (Fig. 3b,c, Supplementary Table S4). One such gene (*pab-1)* encoded a conserved AGO interactor^19^ (Fig. 3b, Supplementary Table S4) and five genes including *C04E6.11, ech-5*, and *rbd-1,* which code for let-7 interactors (Fig. 3c, Supplementary Table S4). RNAi of *cey-1* suppressed the bursting (Fig. 3c, Supplementary Table S4). Knockdown of *ifg-1* also mildly suppressed *let-7(n2853)* vulval bursting from 30 to 6% (Fig. 3b, Supplementary Table S4), although the suppression did not reach a statistically significant level (Anova p-value = 0.078). RNAi knockdown of these genes in the wild type background did not result in vulval bursting, suggesting that these genes do not play a central role in gene regulation, only revealing the function in the sensitized *let-7(n2853)* background (Fig. 3b,c). We cannot, however, rule out the possibility that RNAi knockdown in wild type background may have been ineffective. Overall, these findings support our hypothesis that the identified let-7 physical interactors play a role in let-7-mediated gene repression.

Since dysregulation of *let-7* target gene *lin-41* in vulval-uterine system is sufficient to cause vulval rupturing^49^, we wanted to determine how depletion of putative physical and genetic *let-7* interactors affects *lin-41* expression in the relevant cells. To do this, we used an established *let-7-lin-41* reporter system^49^. We performed RNAi knockdown of genes that enhance (*ech-5*, *rbd-1*, *C04E6.11,* and *let-363*) and suppress (*cey-1*) *let-7(n2853)* vulval bursting in the background of two reporter strains: *pdpy-30::gfp::lin-41 3ʹUTR* and *pdpy-30::gfp::lin-41 3*ʹ*UTR; let-7(n2853)*^49^. RNAi depletion of these genes did not alter *pdpy-30::gfp::lin-41 3*ʹ*UTR* reporter levels in the wildtype background (Fig. 3d,e, Supplementary Table S5), suggesting that these genes do not have a major effect on *lin-41* levels on their own. However, in *let-7(n2853)* background at 15 °C, knockdown of *ech-5* or *rbd-1* substantially increased *lin-41* levels, while *cey-1* depletion reduced *pdpy-30::gfp::lin-41 3*ʹ*UTR* reporter levels in the vulval cells (Fig. 3d,e, Supplementary Table S5). RNAi of *let-363* led to a mild increase in the reporter levels in *let-7(n2853)* at 15 °C, although the increase was not statistically significant (Fig. 3e, Supplementary Table S5). Interestingly, *let-363* depletion was previously reported to increase levels of let-7 target reporters *hbl-1p::gfp::hbl-1* in VNC and *col-10::gfp::lin-41 3′UTR* in hypodermal cells^50^. These observations suggest that *ech-5, rbd-1, cey-1* and perhaps even *let-363* may be contributing to regulation of vulval bursting by modulating *let-7* miRNA activity.

#### RNAi knockdown of genes of let-7 and ALG-1 interactors alters mir-48 mir-241(nDf51) mutant phenotype

**Figure 4:**
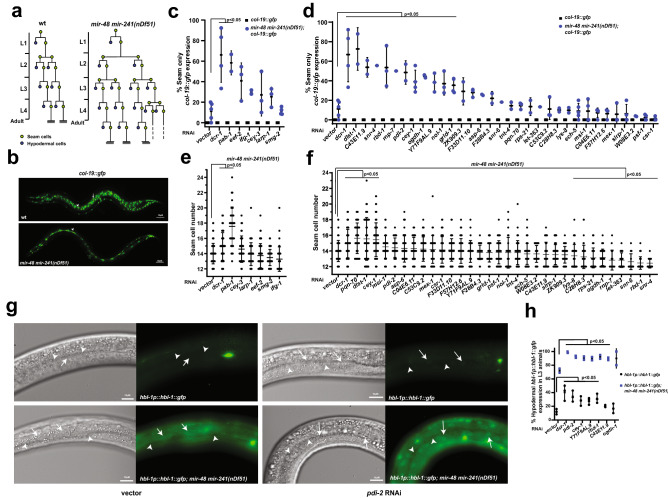
RNAi knockdown of genes encoding for putative ALG-1 and miRNA interactors modulates *mir-48 mir-241(nDf51)* seam cell lineage and hypodermal cell fate defects. (**a**) A schematic representation of seam cell lineages of wild type and *mir-48 mir-241(nDf51)* mutant animals throughout *C. elegans* larval development. (**b**) Wild type young adult animals express adult cell fate marker, *col-19::gfp,* in seam and hypodermal cells, while *mir-48 mir-241(nDf51)* mutants show lack the *col-19::gfp* expression in the hypodermis as young adults some of the time (quantified in c). (**c**,**d**) Effects of RNAi knockdown of genes of (**c**) conserved AGO^19^ and (**d**) other miRISC interactors on hypodermal *col-19::gfp* expression in wild type and *mir-48 mir-241(nDf51)* mutants. Each dot represents an independent RNAi experiment. (**e**,**f**) Effects of RNAi knockdown of genes of (**e**) conserved AGO^19^ and (**f**) other miRISC interactors on the seam cell number in *mir-48 mir-241(nDf51)* mutant young adults*.* Each dot represents seam cell number in an individual worm. Statistical significance was determined by One-way ANOVA with post hoc Bonferroni correction. (**g**) Expression of *hbl-1::gfp::hbl-1* 3′ *UTR* reporter in control and *pdi-2* RNAi in wild type and *mir-48 mir-241(nDf51)* L3 larvae. Arrows indicate hypodermal cells and arrowheads indicate seam cells. (**h**) RNAi of most genes that genetically modified heterochronic defects in *mir-48 mir-241(nDf51)* mutants significantly affected *hbl-1::gfp::hbl-1* 3′ UTR reporter expression in both wildtype and *mir-48 mir-241(nDf51)* backgrounds. Each dot represents an independent RNAi experiment. T-test was used to determine statistical significance*.* Vector = empty vector RNAi control.

We next asked whether miRNA and ALG-1 interactors might functionally coordinate with other members of the *let-7* family of miRNAs. *let-7* family members *mir-48, mir-84* and *mir-241* specify developmental timing in *C. elegans*, regulating seam cell divisions and contributing to L2–L3 larval developmental transition^51^ (Fig. 4a). Deletion of all three miRNAs (*mir-48 mir-241(nDf51); mir-84(n4037)*) results in reiteration of L2 stage seam cell divisions, resulting in an increased number of seam cells and delayed terminal cell differentiation in young adults^51^ (Fig. 4a). Partial deletion (*mir-48 mir-241(nDf51)*) mutants display incompletely penetrant heterochronic phenotype which can be monitored using an adult stage marker, *col-19::gfp,* expressed in seam and hypodermal cells (Fig. 4b). RNAi of 11 genes enhanced the abnormal *col-19::gfp* expression in hypodermal cells (Fig. 4c,d, Supplementary Table S4), with five genes, *pab-1, dlst-1, C43E11.9, snr-4,* and *rbd-1*, enhancing the phenotype to > 50% (Fig. 4c,d Supplementary Table S4). This suggests that these potential miRNA interactors are required for regulation of developmental timing programs. We also examined the effects of gene knockdown on seam cell number. RNAi of three genes, *pqn-70, dlst-1*, and *cey-1*, increased the seam cell number of *mir-48 mir-241(nDf51)* mutants (Fig. 4f, Supplementary Table S4). RNAi of nine genes suppressed seam cell lineage defect, with knockdown of *let-363*, *rbd-1*, *snr-6,* and *snr-4,* restoring the seam cell number to an average of 13 or lower (Fig. 4e,f, Supplementary Table S4). We should note that while most genes were assayed across a minimum of two independent RNAi experiments, four genes (*snr-6*, *snr-4*, *let-363,* and *rnp-7)* were tested only once, as knockdown of these genes caused lethality, reduced brood size, and slowed growth. Depletion of some genes had varied effects on hypodermal *col-19::gfp* expression and seam cell lineage. Knockdown of *C43E11.9, pdi-2,* and *Y71F9AL.9* modified *col-19::gfp* expression but not seam cell number, while knockdown of *pqn-70* and *C28H8.3* modified the seam cell number of *mir-48 mir-241(nDf51)* animals without affecting hypodermal *col-19::gfp* expression (Fig. 4d,f). This could perhaps be explained by distinct roles these genes may play during proliferative seam cell divisions and terminal hypodermal cell fate specification.

Since *let-7* family miRNAs promote L3 cell fates by repressing *hbl-1*^51^, we sought to determine whether the genes that affect heterochronic phenotypes in *mir-48 mir-241*(*nDf51*) background do so by regulating *hbl-1*. We used the *hbl-1p::gfp::hbl-1 3′UTR* fusion construct as a reporter to assess the effects of gene knockdown on levels of HBL-1^52^. Strong *hbl-1* expression can be seen during embryogenesis with hypodermal *hbl-1::gfp::hbl-1 3′UTR* expression decreasing beyond detection at the L3 stage^52^ (Fig. 4g). RNAi knockdown of *pdi-2, rbd-1,Y71F9AL.9,* and *cey-1* resulted in higher percentages of L3 animals expressing the reporter in the wild type background, potentially indicating a miRNA-independent effect (Fig. 4h, Supplementary Table S5). Since depletion of these genes did not affect *col-19:gfp* expression or other heterochronic defects in the wild type (Fig. 4d), perhaps the extent of *hbl-1* derepression was not strong enough to impact developmental timing. This notion is supported by the observation that *hbl-1* reporter expression in *mir-48 mir-241*(*nDf51*) background is observed at a higher rate (75%, Fig. 4h). Knockdown of enhancers of *mir-48 mir-241*(*nDf51*) mutant phenotype derepressed *hbl-1* reporter expression in the *mir-48 mir-241*(*nDf51*) background (Fig. 4h, Supplementary Table S5). Overall, these findings suggest that our proteomics approach captured proteins that may co-ordinate with *let-7* family miRNAs to repress their target, *hbl-1,* and ultimately coordinate developmental timing*.*

#### Depletion of ALG-1 physical interactors altered lsy-6(ot150) phenotype

**Figure 5:**
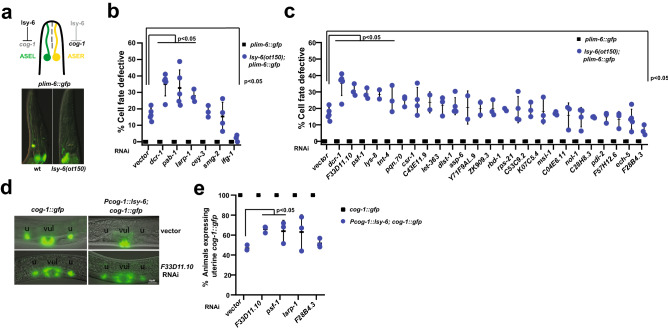
ALG-1 and miRNA interactors genetically interact with *lsy-6(ot150)*. (**a**) *lsy-6* determines the asymmetric expression of chemoreceptors in ASER and ASEL neurons, with *plim-6::gfp* expression marking ASEL neuronal cell fate. ASEL cell defective phenotype in the *lsy-6(ot150)* animals can be observed by lack of *plim-6::gfp* expression some of the time (quantified in b). RNAi knockdown of (**b**) conserved AGO^19^ and (**c**) miRNA/ALG-1 interactors in *plim-6::gfp* or *lsy-6(ot150); plim-6::gfp* animals*.* Each dot represents an independent RNAi experiment. Statistical significance was determined by One-way ANOVA with post hoc Bonferroni correction. (**d**) *cog-1* expression in uterine cells is repressed by *pcog-1::lsy-6*. RNAi of F33D11.10 depresses *cog-1::gfp* in that background (quantified in e). (**e**) RNAi of genes that modify *lsy-6(ot150)* phenotype alleviates lsy-6-mediated repression of *cog-1::gfp* in uterine cells. Each dot represents an independent RNAi experiment. T-test was used to determine statistical significance. Vector = empty vector RNAi control.

We hypothesized that some of the ALG-1 and/or miRNA putative physical interactors could be factors that are generally required for miRISC activity. To test this, we RNAi depleted them in *lsy-6(ot150)* background*. lsy-6* is essential for cell fate determination of chemosensory ASE neurons^53^. lsy-6 represses an ASER cell fate promoting transcription factor *cog-1*, leading to an ASEL neuronal specific gene expression pattern. Loss of *lsy-6* leads to dysregulated gene expression of *cog-1* and downstream effectors resulting in defective ASEL cell fate which leads to lack of *plim-6::gfp* reporter. However, the reduction of function mutant *lsy-6(ot150)* shows partially penetrant cell fate defective phenotype in approximately 20% of animals^53^ (Fig. 5a). Knockdown of 8 genes modified *lsy-6(ot150)* defective phenotype (Fig. 5b,c, Supplementary Table S4) including previously reported conserved AGO interactors *pab-1,* and *larp-1*^19^ (Fig. 5b). Interestingly, we identified two suppressors (*ifg-1,* and *F28B4.3*) of ASEL cell fate defect (Fig. 5b,c, Supplementary Table S4). To determine whether these candidate factors can influence ASEL cell fate independent of lsy-6 miRNA, we knocked them down in wild-type worms and observed no change in *plim-6::gfp* reporter expression in ASEL cells (Fig. 5b,c)*.*

We next determined whether genes that modified *lsy-6* phenotype upon knockdown were important for lsy-6 mediated target activity using *cog-1* reporter system^54^ (Fig. 5d). *cog-1* is expressed in vulval and uterine cells where lsy-6 is absent (Fig. 5d, top left panel). When *lsy-6* is ectopically expressed in these tissues under *cog-1* promoter, there is reduced expression of *cog-1* as a result of lsy-6 mediated repression^54^ (Fig. 5d, top right panel). We performed RNAi knockdown of top hits from lsy-6 assay in the *cog-1* reporter strain and observed no difference in *cog-1* expression (Fig. 5e, Supplementary Table S5), suggesting that these factors do not regulate *cog-1* directly in uterine cells. RNAi knockdown of *F33D11.10* and *psf-1* (enhancers of *lsy-6(ot150)* phenotype) restored *cog-1* expression in the presence of *lsy-6,* suggesting their requirement for lsy-6 mediated *cog-1* repression (Fig. 5e). Knockdown of *larp-1* did not restore *cog-1* expression to a statistically significant level (Fig. 5e, Supplementary Table S5). This could be due to RNAi variability among replicates, or possibly because lsy-6 activity was initially assessed in ASE neurons, while *cog-1* reporter expression was assessed in uterine tissue. Previously reported tissue-specific composition of miRNA-centered complexes and distinct mechanisms of target suppression^44^ support this potential explanation for the observed discrepancy in *larp-1* effects. *lsy-6(ot150)* and *cog-1* reporter assays collectively demonstrate that we identified factors that may directly or indirectly coordinate with lsy-6, affecting its target *cog-1* expression.

Overall, depletion of miRNA complex interactors did not produce a phenotype in the absence of the sensitized miRNA mutations (Figs. 3b,c, 4c,d, 5b,c). While we cannot rule out inefficient RNAi knockdown as a possible explanation, we hypothesize that the tested factors are not critical for regulation of miRNA target gene expression, but rather play a modulatory role in miRNA production and/or activity, or influence gene expression downstream of miRNA activity.

## Discussion

**Figure 6:**
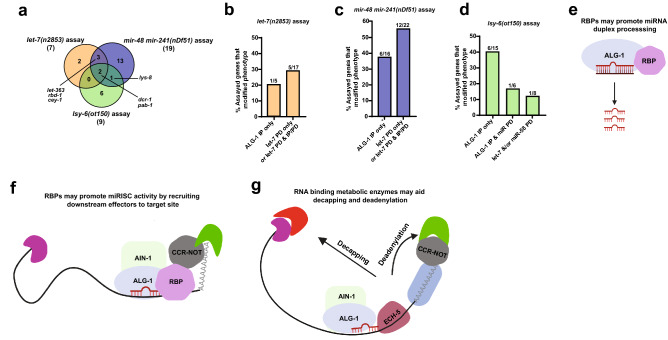
Summary of functionally relevant interactors and models for possible mechanisms through which miRNA and ALG-1 interactors affect gene regulation. (**a**) Venn diagram showing the overlap of functional hits among the three functional assays. (**b**–**d**) Percentages of assayed factors that modulated phenotypes upon their respective gene knockdowns in (**b**) *let-7(n2853)*, (**c**) *mir-48 mir-241(nDf51)*, and (**d**) *lsy-6(ot150)* assays, with protein physical interaction status shown on the x-axis. *One ALG-1 interactor was also identified in miR-2 pulldown in addition to ALG-1 IP. The number of genes that modulated miRNA phenotypes over total number of genes tested is shown above respective bars. (**e**–**f**) RNA binding proteins found interacting with ALG-1 as well as miRNAs could be involved in (**e**) miRNA duplex processing and/or (**f**) facilitating miRISC activity on the targets by associating with downstream effectors. (**g**) Metabolic enzymes with putative RNA binding ability, such as ECH-5, could bind target mRNAs and promote miRISC activity by recruiting deadenylases and decapping proteins. Panels (**e**,**f**), and (**g**) were drawn using BioRender (https://biorender.com).

To better understand miRNA mediated gene regulation, we performed miRNA pulldowns to identify components of miRNA-centered complexes. Our proteomics approach captured 211 miRNA-interacting proteins, some of which were previously reported to precipitate with other miRISC components (Supplementary Table S2). Knockdown of 25 out of 39 genes significantly modulated miRNA mutant phenotypes in one or more assays, suggesting that our pulldowns captured proteins that coordinate with miRNAs to affect gene regulation (Fig. 6a, Supplementary Table S4). Of the 25 hits, knockdown of five genes (*pab-1, let-363, rbd-1, cey-1,* and *lys-8*) and *dcr-1,* a positive control*,* consistently modified miRNA phenotypes in two or more assays (Fig. 6a, Supplementary Table S4). Of the 22 candidate genes tested, RNAi of six genes modulated *let-7(n2853)* vulval bursting phenotype (Fig. 6a,b, Supplementary Table S4). Five of these functional interactors were identified in let-7 PD experiments, either in let-7 PD alone or in let-7 PD plus additional precipitation experiments (Fig. 6b, Supplementary Table S4), suggesting that let-7 interacting factors indeed functionally coordinate with let-7 activity. Knockdown of 16 putative interactors did not modify vulval bursting phenotype of *let-7(n2853)* (Fig. 6b), perhaps due to tissue or time specific physical interactions of these proteins with let-7 miRNA or ALG-1 complexes. Such spatio-temporal complex compositions could explain the corresponding lack of activity in vulval tissue. We cannot, however, rule out insufficient RNAi knockdown or non-specific interactions of these proteins with anti-let-7 oligonucleotide. Knockdown of 18/38 candidate genes genetically modified hypodermal and/or seam cell lineage defects of *mir-48 mir-241(nDf51)* mutants (Fig. 6c, Supplementary Table S4). 12 of these factors were identified in let-7 pulldowns from wild type and/or *mir-48 mir-241; mir-84* mutant backgrounds, suggesting that let-7 PD proteomics captured factors that support let-7 family miRNA activity in developmental timing.

As *lsy-6* miRNA activity is highly localized and unrelated to miRNAs precipitated in our PD experiments, *lsy-6(ot150)* mutation provided a convenient genetic background to identify which factors may be broadly involved miRNA-mediated gene regulation. Of the eight functional hits from the *lsy-6(ot150)* assay, seven factors were identified as ALG-1 interactors (Fig. 6d, Supplementary Table S4), consistent with the idea that ALG-1 IP perhaps precipitated proteins with broad specificities. Lack of *lsy-6(ot150)* phenotype modification by knockdown of let-7 and/or miR-58-associated proteins suggests that miRNA-centered complexes may be unique to the specific miRNAs, possibly due to distinct spatial or temporal expression patterns.

Several genes that modified *let-7(n2853)* vulval bursting in our study, *cey-1*, *ifg-1*, *pab-1,* and *rbd-1* (Fig. 3b,c, Supplemental Table S4), were previously tested in an RNAi screen for suppressors of *let-7(n2853)* vulval bursting, aimed at identifying let-7 target genes^55^. We observed multiple differences between the results of our RNAi screen, performed at the permissive temperature of 15 °C and the previous work, performed at non-permissive 25 °C^55^, which eliminates *let-7* activity. For example, *rbd-1* knockdown enhanced vulval bursting at 15 °C (Fig. 3c, Supplementary Table S4), while it suppressed bursting at 25 °C^55^, suggesting that *rbd-1* may have both let-7 dependent and independent functions. Knockdown of *cey-1* suppressed *let-7(n2853)* vulval bursting at 15 °C in our study (Fig. 3c, Supplementary Table S4), however, no *let-7(n2853)* suppression was observed at 25 °C upon *cey-1* knockdown^55^. These observations suggest that *cey-1* may coordinate with let-7 in target mRNA regulation. Direct comparisons across RNAi studies performed under different conditions can be difficult to interpret and further explorations will be needed to understand the roles of these genes in *let-7-*mediated regulation of gene expression.

How could these putative physical miRNA interactors be coordinating with miRNAs to regulate gene expression? The factors identified in this study could be acting via multiple mechanisms to affect miRNA mutant phenotypes. Some of the miRNA interactors identified in this study have wide-ranging roles in regulation of gene expression. Thus, their knockdown could modify the miRNA reduction-of-function phenotypes directly through miRNA regulation and/or indirectly through regulation of mRNA lifecycle. For example, *pab-1*, a poly(A) binding protein and a homolog of human PABPC1^56^, has well established roles in regulating the stability of mRNA transcripts by affecting translation initiation and mRNA stabilization and decay. PAB-1 has been previously shown to interact with miRISC^19,56^ and to aid miRNA-mediated deadenylation^56^. The enhancement of miRNA reduction-of-function phenotypes upon *pab-1* knockdown may therefore be a result of miRNA-dependent and/or independent functions of *pab-1,* perhaps through loss of target mRNA deadenylation and subsequent mRNA stabilization.

We used the biological and molecular functions predicted by GO term analysis to consider the possible mode of action for the identified miRNA and miRISC interactors. RNA binding proteins were among the classes of genes enriched in our pulldowns (Fig. 2a–h, Supplementary Table S3). Through functional assays, we identified nine interactors with predicted and/or experimentally validated RNA binding activity as genetic interactors of miRNA mutants (Supplementary Table S4). Interestingly, knockdown of genes encoding all nine RNA binding proteins enhanced miRNA mutant phenotypes, consistent with the recent finding that 3’UTR-binding RBPs generally promote miRISC targeting^57^. Some of these RBPs could play a role in miRNA processing (Fig. 6e), some RBPs could potentially facilitate miRISC targeting or activity (Fig. 6f), while other RBPs could regulate localization and stability of miRNAs and/or miRNA targets, ultimately affecting gene regulation.

Translation regulators were also captured in miRNA pulldowns and ALG-1 IP (Supplementary Tables S1, S3), with two of them modifying miRNA phenotypes. Depletion of *ifg-1,* encoding translation initiation factor 4G (eIF4G)^58^, suppressed the ASEL cell fate defect of *lsy-6(ot150)* (Fig. 5b). Given the potential physical association of IFG-1 with ALG-1^19^, it is possible that IFG-1 and miRNAs share common targets; with loss of IFG-1 reducing translation through loss of initiation, thereby suppressing target mRNA overexpression in miRNA reduction of function mutants. RNA helicase F33D11.10 co-precipitated in let-7 pulldown (Supplementary Table S1) and was previously identified as an interactor of ALG-1^19^. Loss of *F33D11.10* activity enhanced the ASEL cell fate defect of *lsy-6(ot150)* mutant (Fig. 5c). RNA helicases have been previously implicated in miRNA processing as well as miRISC activity^59^ and F33D11.10 may be similarly involved in either facilitating miRNA processing, miRISC activity, or both.

A surprising category of interactors identified in our study was the intermediary metabolic enzymes. RNAi depletion of metabolic enzymes DLST-1, OGDH-1 and ECH-5 modified miRNA mutant phenotypes in our study (Figs. 3c, 4d,f, Supplementary Table S4). Several reports have suggested that some metabolic enzymes possess RNA binding functions, previously unidentified due to a lack of conventional RNA binding domains^60–62^. It is possible that the metabolic enzymes identified in our study possess similar dual roles. For instance, *ech-5* encodes a homolog of human AU RNA binding methylglutaconyl-CoA hydratase (AUH)^63^. In humans, AUH plays a dual role as a hydratase and as an RBP, binding AU-rich elements in the 3′ UTR of mRNAs^64^. Other ARE-binding proteins have been previously shown to aid in rapid degradation through deadenylation^65^. In our study, ECH-5 co-precipitated with let-7 miRNAs and *ech-5* depletion enhanced *let-7(n2853)* vulval bursting phenotype (Fig. 3c, Supplementary Table S1). Thus, we might speculate ECH-5 could bridge miRISC complex interaction with deadenylation machinery, with loss of *ech-5* exacerbating the target mRNA stabilization in miRNA mutant backgrounds (Fig. 6g). How other metabolic genes such as *dlst-1* and *ogdh-1*, key players of TCA cycle^66,67^, influence gene regulation remains unclear. Thorough investigations into molecular mechanisms by which these factors coordinate with miRNAs in gene regulation will be needed.

Interestingly, LET-363, *C. elegans* mTOR was identified as an interactor of all three miRNAs in this study (Fig. 1f, Supplementary Table S1). RNAi of *let-363* enhanced the vulval bursting of *let-7(n2853)* and suppressed the seam cell lineage defect of *mir-48 mir-241(nDf51)* mutant (Figs. 3c, 4f, Supplemental Table S4). While we did not test for functional *let-363* requirement in our *lsy-6(ot150)* assay, RNAi of *let-363* was previously reported to exacerbate the cell fate specification defect of *lsy-6(ot150)*^50^*,* consistent with a *let-363* role in miRNA-mediated gene regulation. mTOR activation has been reported to downregulate miRNA biogenesis through Mdm2-mediated DROSHA degradation in mice^68^. However, the physical association of LET-363 with miRNAs was surprising. If confirmed, these persistent physical and functional interactions of LET-363 with miRNA-centered complexes should be further explored to establish the mechanistic connection between mTOR and miRNA-mediated gene regulatory activity.

Do miRNAs within the same family associate with same set of protein interactors? *let-7* family miRNAs are well-studied in *C. elegans*. The four most abundant members of the *let-7* family, *let-7, mir-48, mir-84* and *mir-241,* are crucial components of the heterochronic pathway, regulating cell fates during larval development^32,53^. The miRNAs are thought to function semi-redundantly, with distinct targeting capabilities^69^. Part of their ability to target unique targets could come from discrete protein interactors adding a layer of specificity between a *let-7* family miRNA and its target. Yet not much is known about the protein interacting partners of individual members of this family. By performing pulldowns with a let-7 specific oligo from wild type and *mir-48 mir-241(nDf51); mir-84(n4037)* background, we began the task of unraveling which interactors may be specific to let-7 itself or other family members (Fig. 1h, Supplementary Table S1). For example, ECH-5 and C04E6.11 were identified in let-7 pulldowns from both genetic backgrounds, suggesting that they most likely interact with let-7 (Supplementary Table S1). RNAi depletion of *ech-5* or *C04E6.11* enhanced the *let-7(n2853)* phenotype but showed no effect on the *mir-48 mir-241(nDf51)* associated phenotypes (Figs. 3c, 4d). Thus, it is possible that ECH-5 and C04E6.11 specifically interact with, and provide functional support for, let-7 itself. CEY-1 was captured in let-7 pulldowns from both wild type and *mir-48 mir-241; mir-84* mutant background, but depletion of *cey-1* suppressed *let-7(n2853)* vulval bursting and enhanced *mir-48 mir-241(nDf51)* mutant phenotypes (Figs. 3c, 4d). This suggests that *cey-1* may functionally interact with multiple members of the *let-7* family, potentially through distinct mechanisms. Previously, miR-241 complementary oligo pulldown captured CEY-1^44^, although it remains difficult to assess specificity, as miR-241 oligo may capture other members of the family, similar to let-7^47^. Overall, we cannot rule out the possibility that some of the factors precipitating in miRNA pulldowns are non-specifically interacting with the precipitating oligo, rather than with the miRNA-centered complexes. It is also possible that let-7 interactions may be altered in the *mir-48 mir-241(nDf51); mir-84(n4037)* background. Similarly, the severe developmental timing defect of *mir-48 mir-241(nDf51); mir-84(n4037)* animals could hinder identification of *bona fide* interactors of let-7 in that background. However, the high rate of functional relevance of these factors for miRNA-mediated gene regulation suggests that this approach captures auxiliary factors that may coordinate with miRNAs mediated gene regulation.

## Supplementary Information

Supplementary Table S5.

Supplementary Table S1.

Supplementary Table S2.

Supplementary Table S3.

Supplementary Table S4.
